# Supplementary material for: Lead Isotopic Constraints on the Provenance of Antarctic Dust and Atmospheric Circulation Patterns Prior to the Mid-Brunhes Event (~430 kyr ago)
Source: Molecules. 2022 Jun 30;27(13):4208. doi: 10.3390/molecules27134208 (PMC9268056; doi:10.3390/molecules27134208)
Supplement: Supplementary file 1 [file molecules-27-04208-s001.zip › molecules-1773281-supplementary.pdf]

Supplementary Materials

Lead Isotopic Constraints on the Provenance of Antarctic Dust and Atmospheric Circulation Patterns Prior to the Mid-Brunhes Event (~430 kyr ago)

Changhee Han [1,2], Laurie J. Burn [3], Paul Vallelonga [3,4], Soon Do Hur [2], Claude F. Boutron [5], Yeongcheol Han [2], Sanghee Lee [1,6], Ahhyung Lee [1], Sungmin Hong [1,*]

[1] Department of Ocean Sciences, Inha University, 100 Inha-ro, Michuhol-gu, Incheon 22212, Korea
[2] Division of Glacial Environment Research, Korea Polar Research Institute, 26 Songdomirae-ro, Yeonsu-gu, Incheon 21990, Korea
[3] Department of Imaging and Applied Physics, Curtin University of Technology, GPO Box U1987, Perth, WA 6845, Australia
[4] Oceans Graduate School and UWA Oceans Institute, The University of Western Australia, Crawley, WA 6009, Australia
[5] Institut des Géosciences de l'Environnement, Université Grenoble Alpes/CNRS, 54 rue Molière, 38400 Saint Martin d'Hères, France
[6] Research Unit of Frontier Exploration, Korea Polar Research Institute, 26 Songdomirae-ro, Yeonsu-gu, Incheon 21990, Korea

* Corresponding author: smhong@inha.ac.kr; Tel.: +82-32-860-7708; Fax: +82-32-862-5236

Summary

There are 13 pages in this Supplementary Material including 4 tables and 5 figures.

Table S1. Data obtained from 40 depth intervals from 2973.9 (age of 572,800 yr B.P.) to 3189.45 m (age of 801,590 yr B.P.). The depths and estimated ages given in the table are the depth and age for the top of the samples. Uncertainties in the isotope ratios are for 95% confidence intervals.

No.	Depth (m)	Age (Years BP)	δD (‰)	Concentration Pb (pg/g)	Concentration Ba (pg/g)	Concentration In (fg/g)	$^{206}Pb/^{207}Pb$	±	$^{208}Pb/^{207}Pb$	±	Pb/Ba (by wt.)	In/Pb (by wt.)	Dust Pb (%)
1	2973.91	572,800	-403	1.01	19.7	4.6	1.1945	0.0032	2.4674	0.0044	0.05	0.005	58.5
2	2974.08	572,900	-403	0.45	8.8	2.4	1.1945	0.0034	2.4729	0.0057	0.05	0.005	58.1
3	2985.07	576,765	-404	0.72	15.0	4.4	1.1975	0.0028	2.4703	0.0049	0.05	0.006	62.3
4	2990.35	578,965	-398	0.83	15.4	3.6	1.1874	0.0028	2.4618	0.0050	0.05	0.004	55.6
5	2995.92	581,610	-415	1.31	39.5	10.5	1.1995	0.0024	2.4729	0.0041	0.03	0.008	90.3
6	3001.41	585,975	-424	2.14	38.4	15.0	1.1853	0.0027	2.4556	0.0047	0.06	0.007	53.8
7	3001.58	586,225	-424	0.97	20.5	6.5	1.1959	0.0033	2.4744	0.0051	0.05	0.007	63.6
8	3012.41	597,180	-432	6.81	122.3	36.9	1.1975	0.0021	2.4674	0.0032	0.06	0.005	53.9
9	3012.58	597,510	-432	4.30	81.5	18.7	1.1970	0.0022	2.4696	0.0041	0.05	0.004	56.8
10	3023.35	610,680	-401	0.46	5.0	2.1	1.1824	0.0032	2.4663	0.0055	0.09	0.005	32.4
11	3034.35	622,895	-405	2.21	72.4	25.9	1.1958	0.0027	2.4644	0.0047	0.03	0.012	98.1
12	3040.13	631,365	-438	3.84	100.1	22.7	1.2021	0.0022	2.4743	0.0048	0.04	0.006	78.1
13	3045.42	644,485	-433	8.63	161.9	40.1	1.1955	0.0024	2.4686	0.0040	0.05	0.005	56.3
14	3056.48	662,800	-437	19.05	297.1	68.7	1.2041	0.0018	2.4686	0.0037	0.06	0.004	46.8
15	3056.61	663,290	-437	16.99	311.0	58.9	1.2004	0.0020	2.4661	0.0040	0.06	0.003	54.9
16	3072.92	682,755	-425	3.54	40.4	10.8	1.1845	0.0026	2.4576	0.0045	0.09	0.003	34.2
17	3073.08	682,990	-425	2.93	48.7	23.1	1.2011	0.0023	2.4763	0.0037	0.06	0.008	49.9
18	3078.35	687,730	-417	1.94	32.7	7.5	1.1964	0.0019	2.4667	0.0035	0.06	0.004	50.5
19	3083.85	691,890	-411	1.23	15.0	5.1	1.2047	0.0034	2.4636	0.0061	0.08	0.004	36.5
20	3089.42	695,620	-403	3.54	11.4	63.3	1.2332	0.0032	2.4902	0.0047	0.31	0.018	9.7
21	3089.59	695,785	-403	1.63	6.7	10.7	1.2000	0.0030	2.4693	0.0047	0.24	0.007	12.4
22	3094.92	699,080	-405	1.33	21.0	15.2	1.2119	0.0036	2.4748	0.0045	0.06	0.011	47.2

23	3095.09	699,240	-405	2.15	9.9	18.6	1.2086	0.0029	2.4703	0.0043	0.22	0.009	13.8
24	3100.42	702,580	-408	0.84	9.5	2.5	1.1907	0.0023	2.4658	0.0035	0.09	0.003	33.8
25	3100.57	702,740	-408	13.21	13.0	3.9	1.1900	0.0022	2.4579	0.0042	1.02	0.0003	2.9
26	3106.08	706,315	-407	0.70	14.5	8.0	1.2044	0.0024	2.4767	0.0046	0.05	0.011	62.1
27	3111.35	711,860	-412	6.97	171.4	74.6	1.2299	0.0020	2.4853	0.0033	0.04	0.011	73.8
28	3122.35	721,290	-438	9.90	88.3	17.9	1.2259	0.0017	2.4751	0.0032	0.11	0.002	26.8
29	3127.85	727,415	-422	4.06	36.3	117.0	1.2307	0.0024	2.4939	0.0037	0.11	0.029	26.9
30	3133.45	732,980	-412	1.27	30.7	5.8	1.2013	0.0020	2.4765	0.0032	0.04	0.005	72.8
31	3133.60	733,260	-412	2.11	31.9	16.2	1.2137	0.0022	2.4766	0.0035	0.07	0.008	45.2
32	3139.95	739,730	-439	17.07	453.1	86.4	1.1965	0.0020	2.4713	0.0034	0.04	0.005	79.6
33	3145.45	746,495	-439	7.52	143.6	22.2	1.2108	0.0022	2.4742	0.0039	0.05	0.003	57.3
34	3149.85	751,860	-439	8.64	177.8	35.4	1.1973	0.0018	2.4704	0.0034	0.05	0.004	61.8
35	3156.45	759,970	-429	1.96	37.0	15.0	1.1990	0.0023	2.4758	0.0038	0.05	0.008	56.8
36	3160.85	765,820	-420	2.54	25.6	10.5	1.1877	0.0021	2.4642	0.0033	0.10	0.004	30.3
37	3165.32	771,640	-415	0.89	25.9	4.2	1.1986	0.0041	2.4725	0.0052	0.03	0.005	87.1
38	3172.95	781,015	-403	0.49	10.7	3.8	1.2110	0.0032	2.4849	0.0053	0.05	0.008	66.3
39	3178.52	786,950	-396	1.09	12.9	9.7	1.2013	0.0067	2.4747	0.0089	0.09	0.009	35.3
40	3189.45	801,590	-441	108	627	93	1.2295	0.0015	2.4749	0.0024	0.17	0.001	17.4
All periods		Mean		6.9	85	25	1.2022		2.4715		0.10	0.0067	50.3
		Min-Max		0.45-108	5.0-627	2.1-117	1.1824-1.2332		2.4556-2.4939		0.03-1.02	0.0003-0.029	2.9-98.1

Table S2. Statistics (mean ± standard deviation, SD) that summarize the data. The individual data are separated according to cold, intermediate, and warm climatic conditions and a dust-derived Pb fraction of > 60% (dust-dominant) and < 60% (non-dust dominant) (see text). The post-MBE data are from Vallelonga et al. [1]. The number of samples is given in parentheses.

Period		Samples		δD (‰)	Dust Flux (mg/m^2/yr)	Concentration (pg/g)		$^{206}Pb/^{207}Pb$	$^{208}Pb/^{207}Pb$	Dust Pb (%)
						Pb	Ba			
Post-MBE	Cold	dust-dominant Pb (n=26)	Mean	-438 ± 3.3	6.3 ± 3.1	8.9 ± 4.8	233 ± 117	1.2015 ± 0.0052	2.4699 ± 0.0099	81.4 ± 12.7
			Min-Max	-444.2 ~ -431.9	2.3 ~ 13	2.1 ~ 19	71 ~ 467	1.1931 ~ 1.2142	2.4445 ~ 2.4900	60.7 ~ 100
		non-dust dominant Pb (n=5)	Mean	-437 ± 2.8	4.2 ± 1.2	13 ± 10	151 ± 62	1.2231 ± 0.0252	2.4836 ± 0.0170	42.1 ± 15.9
			Min-Max	-439.2 ~ -433.7	2.9 ~ 5.7	6.1 ~ 29	82 ~ 240	1.1918 ~ 1.2570	2.4616 ~ 2.5066	17.9 ~ 57.8
	Intermediate	dust-dominant Pb (n=11)	Mean	-420 ± 6.4	1.6 ± 1.2	2.6 ± 4.9	68 ± 123	1.2069 ± 0.0088	2.4772 ± 0.0076	82.0 ± 11.4
			Min-Max	-428.7 ~ -410.9	0.46 ~ 3.8	0.41 ~ 17	13 ~ 436	1.1961 ~ 1.2237	2.4613 ~ 2.4889	60.5 ~ 100
		non-dust dominant Pb (n=15)	Mean	-421 ± 6.2	1.3 ± 1.0	2.9 ± 2.0	40 ± 39	1.2346 ± 0.0117	2.4892 ± 0.0118	41.0 ± 12.0
			Min-Max	-428.7 ~ -411.6	0.31 ~ 4.4	0.83 ~ 9.2	14 ~ 174	1.2143 ~ 1.2541	2.4720 ~ 2.5074	18.8 ~ 59.3
	Warm	dust-dominant Pb (n=15)	Mean	-399 ± 7.4	0.42 ± 0.19	0.28 ± 0.11	8.5 ± 2.8	1.2096 ± 0.0143	2.4818 ± 0.0139	88.7 ± 12.9
			Min-Max	-409.3 ~ -386.6	0.23 ~ 0.97	0.15 ~ 0.52	5.4 ~ 14	1.1844 ~ 1.2302	2.4589 ~ 2.5010	63.2 ~ 100
		non-dust dominant Pb (n=2)	Mean	-407 ± 3.4	0.32 ± 0.05	0.62 ± 0.11	11 ± 3.1	1.2219 ± 0.0031	2.4825 ± 0.0046	52.7 ± 5.3
			Min-Max	-409.3 ~ -404.6	0.29 ~ 0.36	0.54 ~ 0.70	8.8 ~ 13	1.2197 ~ 1.2240	2.4793 ~ 2.4858	48.9 ~ 56.5
Pre-MBE	Cold	dust-dominant Pb (n=3)	Mean	-439 ± 0.2	7.0 ± 4.8	9.8 ± 6.7	244 ± 186	1.1986 ± 0.0031	2.4720 ± 0.0021	73.2 ± 9.9
			Min-Max	-438.7 ~ -438.3	3.2 ~ 12	3.8 ~ 17	100 ~ 453	1.1965 ~ 1.2021	2.4704 ~ 2.4743	61.8 ~ 79.6
		non-dust dominant Pb (n=7)	Mean	-436 ± 3.1	6.0 ± 2.0	10 ± 5.5	172 ± 94	1.2045 ± 0.0108	2.4699 ± 0.0034	50.4 ± 11.0
			Min-Max	-439.2 ~ -432.3	3.7 ~ 9.0	4.3 ~ 19	81 ~ 311	1.1955 ~ 1.2259	2.4661 ~ 2.4751	26.8 ~ 57.3
	Intermediate	dust-dominant Pb (n=5)	Mean	-415 ± 4.8	1.0 ± 0.27	2.3 ± 2.6	58 ± 64	1.2050 ± 0.0140	2.4763 ± 0.0053	77.5 ± 11.0
			Min-Max	-423.5 ~ -411.7	0.63 ~ 1.2	0.89 ~ 7.0	21 ~ 171	1.1959 ~ 1.2299	2.4725 ~ 2.4853	63.6 ~ 90.3
		non-dust dominant Pb (n=9)	Mean	-420 ± 6.2	1.2 ± 0.28	2.5 ± 0.88	34 ± 9.5	1.2003 ± 0.0149	2.4700 ± 0.0119	42.7 ± 10.9
			Min-Max	-429.0 ~ -410.6	0.76 ~ 1.5	1.2 ~ 4.1	15 ~ 49	1.1845 ~ 1.2307	2.4556 ~ 2.4939	26.9 ~ 56.8
	Warm	dust-dominant Pb (n=4)	Mean	-405 ± 1.4	0.70 ± 0.07	1.0 ± 0.79	28 ± 30	1.2022 ± 0.0069	2.4741 ± 0.0088	72.2 ± 17.4
			Min-Max	-406.7 ~ -403.4	0.60 ~ 0.76	0.49 ~ 2.2	11 ~ 72	1.1958 ~ 1.2110	2.4644 ~ 2.4849	62.1 ~ 98.1
		non-dust dominant Pb (n=11)	Mean	-403 ± 3.8	0.61 ± 0.25	2.4 ± 3.7	12 ± 5.0	1.1995 ± 0.0143	2.4701 ± 0.0084	32.7 ± 20.6
			Min-Max	-408 ~ -396.1	0.21 ~ 1.2	0.45 ~ 13	5.0 ~ 21	1.1824 ~ 1.2332	2.4579 ~ 2.4902	2.9 ~ 58.5

Table S3. The relative source strength (in %) of the potential dust sources calculated for dust-dominant samples using an isotope mixing model (see Method).

Period			Patagonia /TdF	Central Western Argentina (CWA)				PAP	Volcanic Contribution
				Total	N-CWA	M-CWA	S-CWA		
Post-MBE	Cold (n=18) [a]	Median ± S.D.	53 ± 25	27 ± 20	5.8 ± 8.1	8.3 ± 3.7	4.8 ± 7.8	4.4 ± 21	12 ± 12
		(min-max)	(3.4-81)	(8.1-44)	(1.3-30)	(1.3-13)	(0.83-28)	(0.81-62)	(0.00-38)
	Intermediate (n=8)	Median ± S.D.	32 ± 18	33 ± 25	13 ± 13	8.4 ± 6.3	8.7 ± 5.4	6.0 ± 2.4	24 ± 9.6
		(min-max)	(13-67)	(5.6-57)	(1.3-37)	(1.7-19)	(2.5-20)	(2.0-9.9)	(6.4-39)
	Warm (n=14)	Median ± S.D.	11 ± 32	42 ± 45	5.3 ± 14	21 ± 24	4.9 ± 6.9	9.9 ± 21	4.0 ± 12
		(min-max)	(0.14-85)	(9.5-94)	(0.87-44)	(3.1-85)	(0.14-24)	(0.86-73)	(0.00-37)
Pre-MBE	Cold (n=2)	Median ± S.D.	63 ± 8.9	12 ± 5.9	2.9 ± 1.1	3.9 ± 1.7	5.4 ± 3.1	3.9 ± 1.9	21 ± 1.1
		(min-max)	(57-69)	(8.0-16)	(2.1-3.7)	(2.7-5.0)	(3.2-7.6)	(2.6-5.3)	(20-22)
	Intermediate (n=3)	Median ± S.D.	57 ± 2.7	20 ± 7.4	4.0 ± 1.7	6.1 ± 2.0	10 ± 3.7	6.7 ± 0.64	13 ± 9.3
		(min-max)	(55-60)	(11-26)	(1.9-5.1)	(3.7-7.8)	(5.7-13)	(6.7-7.8)	(9.7-27)
	Warm (n=3)	Median ± S.D.	58 ± 30	24 ± 17	4.4 ± 5.8	7.4 ± 4.0	4.2 ± 7.4	3.1 ± 13	34 ± 20
		(min-max)	(14-71)	(2.9-27)	(1.3-13)	(0.62-7.8)	(1.0-15)	(0.94-25)	(1.9-38)

[a] number of samples with isotopically feasible solutions as defined by an isotope mixing model.

Table S4. The relative source strength (in %) of the potential volcanic sources calculated for non-dust dominant samples using an isotope mixing model (see Method).

Period			McMurdo Volcanic Group	Andean Volcanic Belt			Easter Island	Dust Contribution
				Total	CVZ	SVZ + AVZ		
Post-MBE	Cold (n=5) [a]	Median ± S.D.	30 ± 25	16 ± 6.8	5.6 ± 3.4	8.4 ± 3.4	8.7 ± 3.1	46 ± 16
		(min-max)	(1.6-66)	(7.8-44)	(2.1-11)	(4.4-33)	(8.0-15)	(18-58)
	Intermediate (n=14)	Median ± S.D.	42 ± 13	12 ± 6.2	4.6 ± 1.7	6.0 ± 4.5	8.4 ± 3.5	39 ± 11
		(min-max)	(19-61)	(3.0-21)	(1.5-7.0)	(1.5-18)	(2.4-14)	(19-57)
	Warm (n=2)	Median ± S.D.	27 ± 5.2	11 ± 2.7	4.1 ± 0.71	6.6 ± 2.0	10 ± 1.1	53 ± 5.3
		(min-max)	(23-30)	(9.8-12)	(3.6-4.6)	(5.2-7.9)	(9.3-11)	(49-56)
Pre-MBE	Cold (n=7)	Median ± S.D.	5.2 ± 11	26 ± 11	4.7 ± 3.9	19 ± 7.5	13 ± 5.4	55 ± 11
		(min-max)	(3.5-33)	(15-36)	(1.6-12)	(10-35)	(3.7-20)	(27-57)
	Intermediate (n=9)	Median ± S.D.	3.4 ± 11	38 ± 28	9.3 ± 7.5	19 ± 20	5.8 ± 7.6	45 ± 11
		(min-max)	(0.28-32)	(19-66)	(0.80-21)	(11-63)	(1.4-20)	(27-57)
	Warm (n=10)	Median ± S.D.	3.8 ± 9.3	38 ± 28	9.5 ± 6.4	27 ± 22	11 ± 14	35 ± 22
		(min-max)	(0.52-30)	(20-91)	(4.2-22)	(13-83)	(4.1-40)	(2.9-59)

[a] number of samples with isotopically feasible solutions as defined by an isotope mixing model.

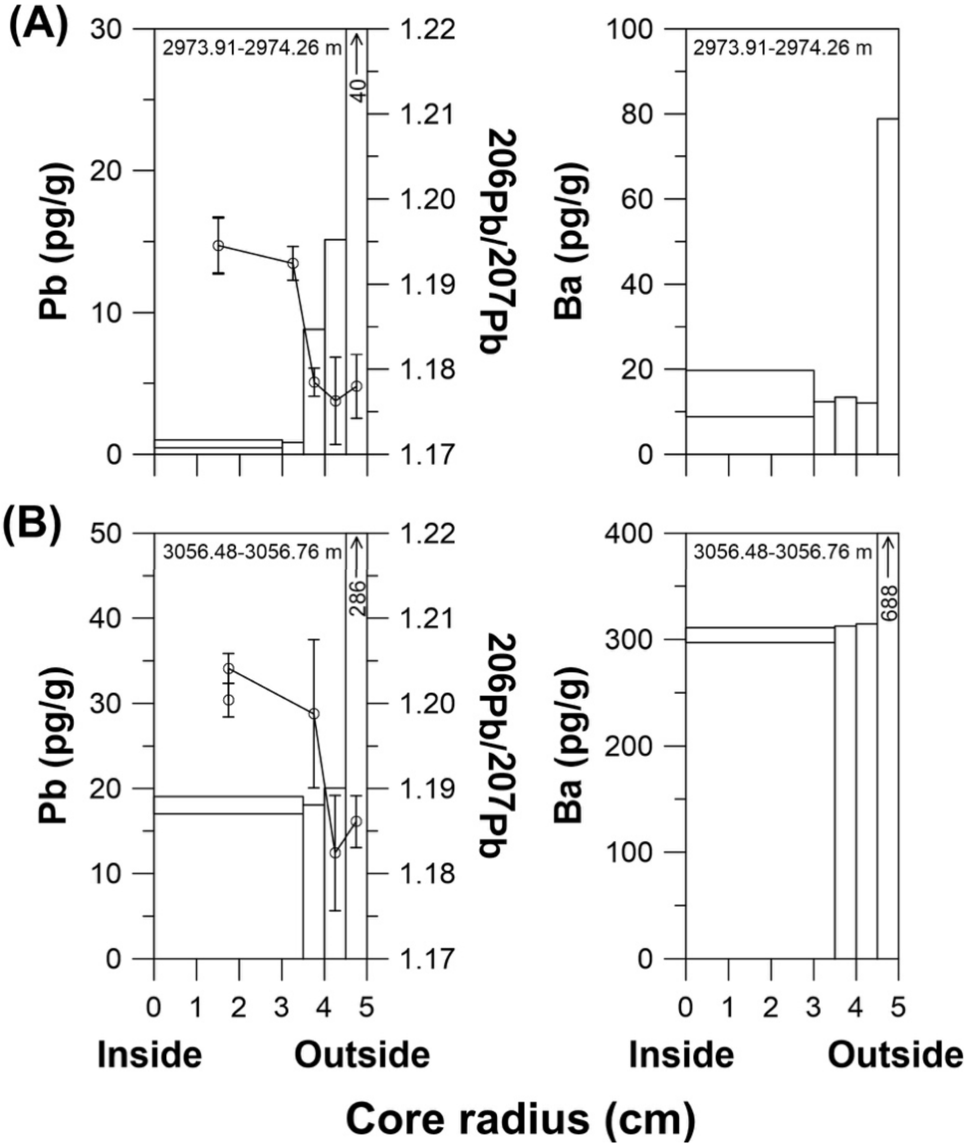

Figure S1. Changes in Pb and Ba concentrations and $^{206}Pb/^{207}Pb$ ratios (open circles) as a function of radius in two sections: (**A**) depth of 2973.91 m (572.8 kyr B.P.), MIS 15.1 interglacial; (**B**) depth of 3056.48 m (662.8 kyr B.P.), MIS 14 glacial. Two different values are given for concentrations and isotope ratios in the innermost part because the inner cores were divided into two consecutive 20 cm long parts. The error bars of the $^{206}Pb/^{207}Pb$ ratios are 95% confidence intervals.

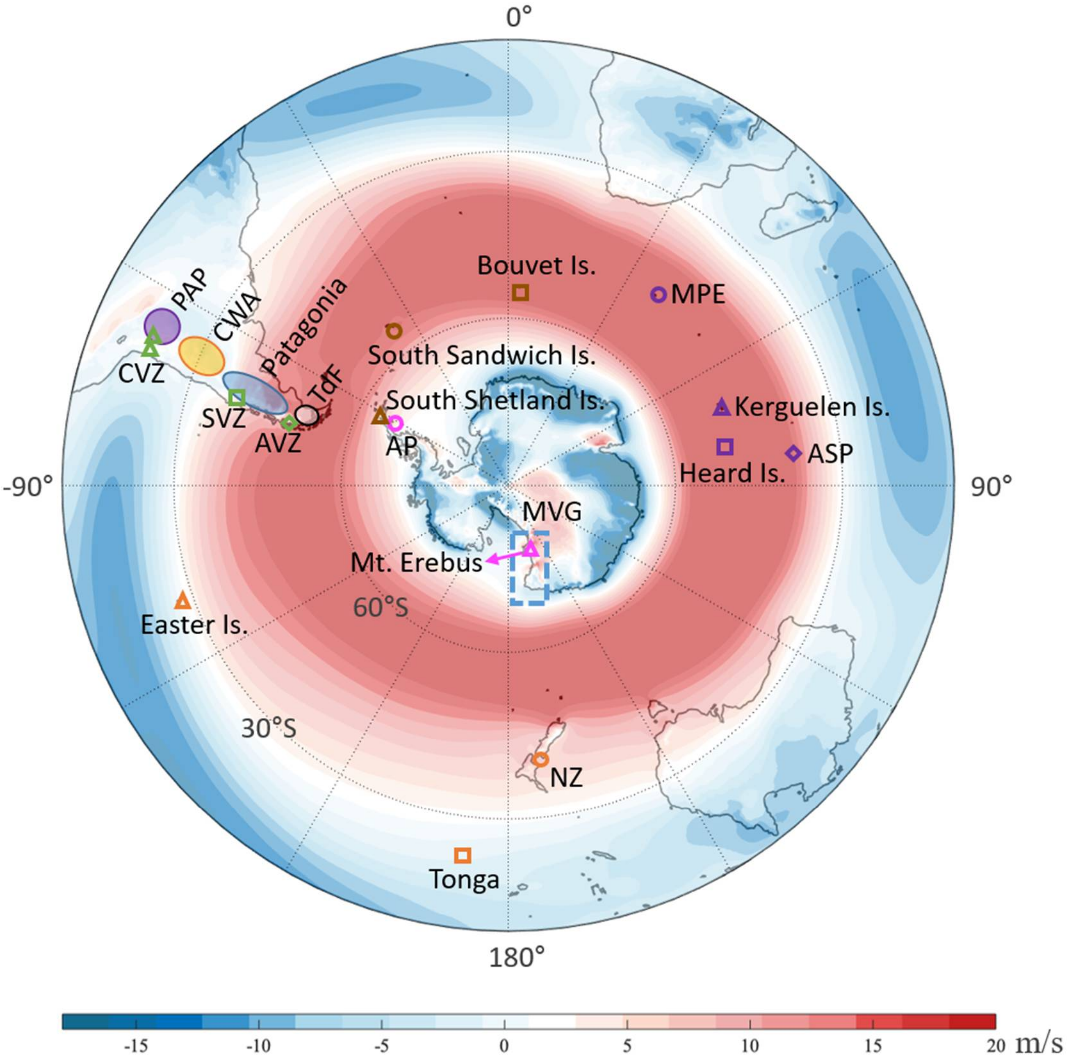

Figure S2. Map of the Potential Source Areas of dust in southern South America and the location of volcanic sources. Shading on this map shows the annual mean 850 hPa westerly wind component obtained from the European Centre for Medium-Range Weather Forecasts (ECMWF) Reanalysis 5 (ERA5; available at: https://www.ecmwf.int/en/forecasts/datasets/reanalysis-datasets/era5). CWA: central-western Argentina. PAP: Puna-Altiplano Plateau. TdF: Tierra del Fuego. ASP: Amsterdam-St. Paul Island. AP: Antarctic Peninsula. CVZ (Central Volcanic Zone), SVZ (Southern Volcanic Zone), and AVZ (Austral Volcanic Zone) of the Andean Volcanic Belt. MPE: Marion and Prince Edward Island. MVG: McMurdo Volcanic Group. NZ: New Zealand.

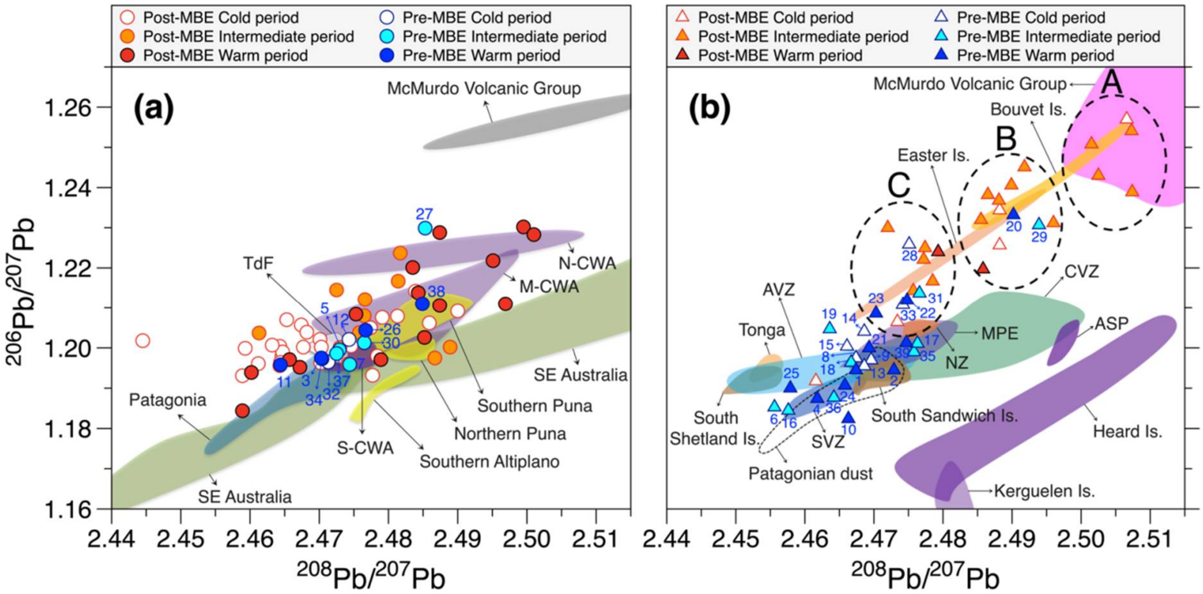

Figure S3. A plot of the $^{206}Pb/^{207}Pb$ versus $^{208}Pb/^{207}Pb$ in the EDC ice core samples showing the identification numbers of pre-MBE in Table S1. Also included are previously published data from the EDC ice core during the reporting period from 2 kyr (MIS 1) to 220 kyr B.P. (MIS 7.3) [1]. The observed data are divided into isotopic compositions for (**a**) dust-dominant samples with a dust-derived Pb fraction of > 60% and (**b**) non-dust dominant samples with a dust-derive Pb fraction of < 60% (see text). Pb isotopic compositions of potential dust sources from published literature: Puna-Altiplano Plateau (PAP) [2], central-western Argentina (CWA) [2], Patagonia/Tierra del Fuego (TdF) [2], southeastern (SE) Australia (Murray-Darling Basin) [1,3]. Pb isotopic compositions defined for volcanic sources are also from published literature (see Figure S4).

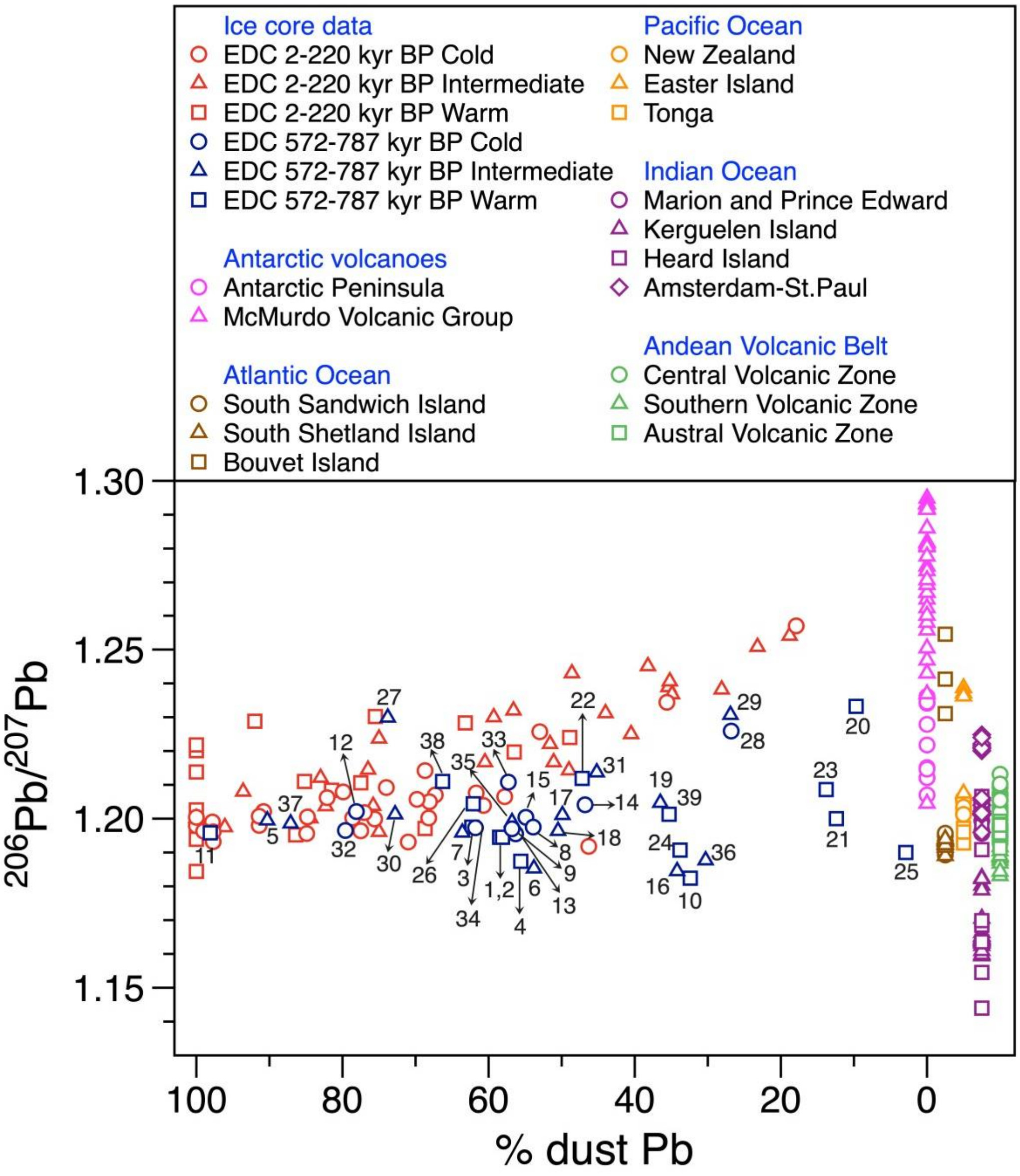

Figure S4. Comparison of $^{206}Pb/^{207}Pb$ ratios between the pre-MBE and post-MBE intervals as a function of dust fraction of Pb showing the identification numbers of pre-MBE samples in Table S1. The end-members of volcanic $^{206}Pb/^{207}Pb$ ratios for the potential volcanic sources from published literature: Antarctic Peninsula basalts [4], McMurdo Volcanic Group [5,6], South Sandwich Island [7], South Shetland Island [8], Bouvet Island [9], New Zealand [10], Easter Island and Tonga [9], Marion and Prince Edward [11], Kerguelen Island [12], Heard Island [13], Amsterdam-St. Paul Island [14], Andean Volcanic Belt [15,16].

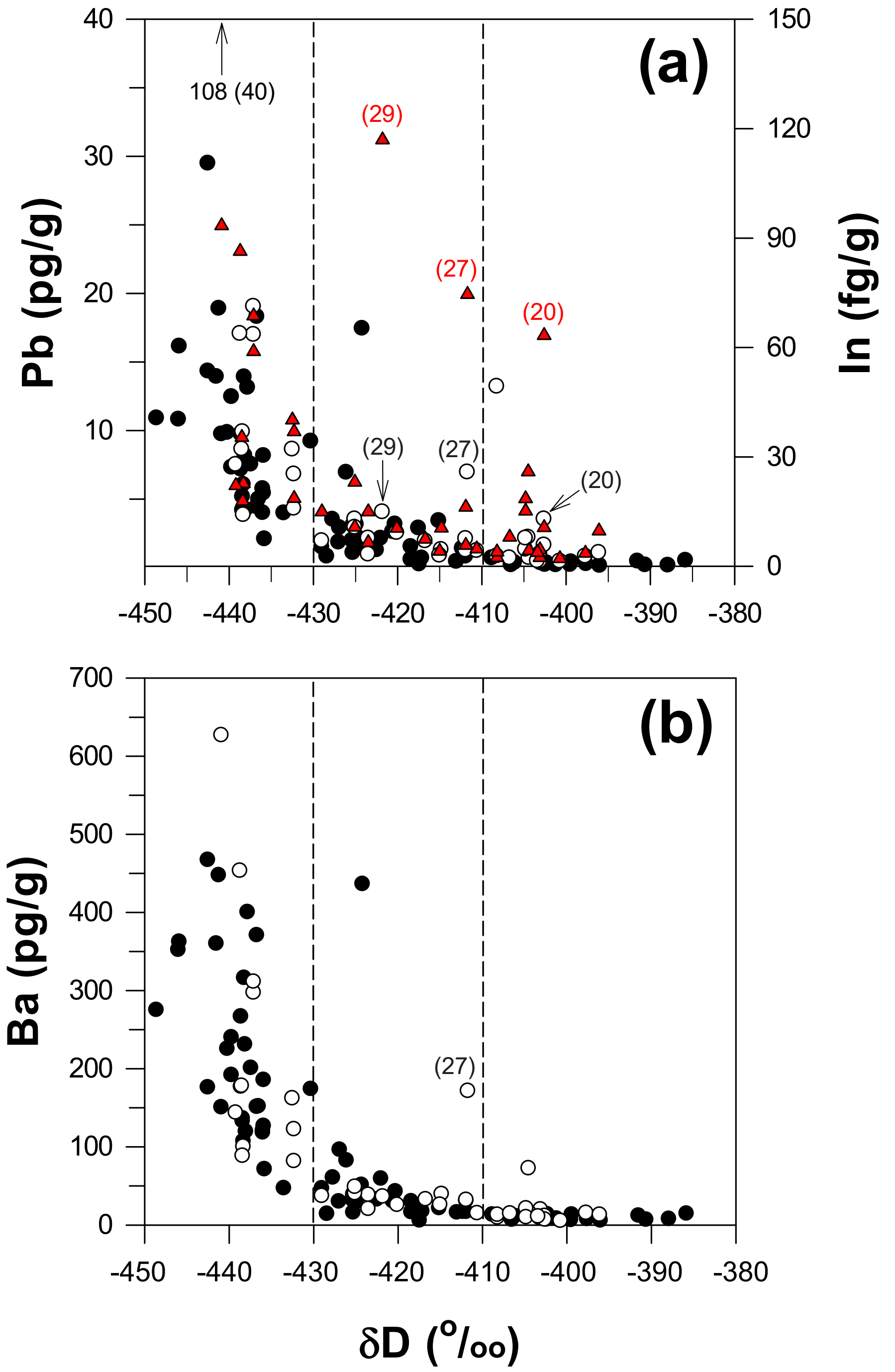

Figure S5. Changes in (**a**) the concentrations of Pb and In as a function of the deuterium content and (**b**) Ba concentrations. Our pre-MBE data are shown as open circles for Pb and Ba and red triangles for In and published post-MBE data [1] as solid circles. The numbers in parentheses represent sample numbers in Table S1 that are mentioned in the text.

References

1. Vallelonga, P.; Gabrielli, P.; Balliana, E.; Wegner, A.; Delmonte, B.; Turetta, C.; Burton, G.; Vanhaecke, F.; Rosman, K.J.R.; Hong, S.; et al. Lead isotopic compositions in the EPICA Dome C ice core and Southern Hemisphere Potential Source Areas. *Quat. Sci. Rev.* **2010**, *29*, 247-255.

2. Gili, S.; Gaiero, D.M.; Goldstein, S.L.; Chemale, F.; Koester, E.; Jweda, J.; Vallelonga, P.; Kaplan, M.R. Provenance of dust to Antarctica: A lead isotopic perspective. *Geophys. Res. Lett.* **2016**, *43*, 2291-2298.

3. Revel-Rolland, M.; De Deckker, P.; Delmonte, B.; Hesse, P.P.; Magee, J.W.; Basile-Doelsch, I.; Grousset, F.; Bosch, D. Eastern Australia: A possible source of dust in East Antarctica interglacial ice. *Earth Planet. Sci. Lett.* **2006**, *249*, 1-13.

4. Hole, M.J.; Kempton, P.D.; Millar, I.L. Trace-element and isotopic characteristics of small-degree melts of the asthenosphere: Evidence from the alkalic basalts of the Antarctic Peninsula. *Chem. Geol.* **1993**, *109*, 51-68.

5. Rocholl, A.; Stein, M.; Molzahn, M.; Hart, S.R.; Worner, G. Geochemical evolution of rift magmas by progressive tapping of a stratified mantle source beneath the Ross Sea Rift, Northern Victoria Land, Antarctica. *Earth Planet. Sci. Lett.* **1995**, *131*, 207-224.

6. Sun, S.S.; Hanson, G.N. Origin of Ross Island basanitoids and limitations upon the heterogeneity of mantle sources for alkali basalts and nephelinites. *Contrib. Mineral. Petrol.* **1975**, *52(2)*, 77-106.

7. Barreiro, B. Lead isotopic compositions of South Sandwich Island volcanic rocks and their bearing on magmagenesis in intra-oceanic island arcs. *Geochim. Cosmochim. Acta* **1983**, *47*, 817-822.

8. Lee, M.J.; Lee, J.I.; Choe, W.H.; Park, C.H. Trace element and isotopic evidence for temporal changes of the mantle sources in the South Shetland Island, Antarctica. *Geochem. J.* **2008**, *42*, 207-219.

9. Sun, S. Lead isotopic study of young volcanic rocks from mid-ocean ridges, ocean islands and island arcs. *Philos. Trans. Royal Soc. A* **1980**, *297*, 409-445.

10. McCulloch, M.T.; Kyser, T.K.; Woodhead, J.D.; Kinsley, L. Pb-Sr-Nd-O isotopic constraints on the origin of rhyolites from the Taupo Volcanic Zone of New Zealand: evidence for assimilation followed by fractionation from basalt. *Contrib. Mineral. Petrol.* **1994**, *115(3)*, 303-312.

11. le Roex, A.P.; Chevallier, L.; Verwoerd, W.J.; Barends, R. Petrology and geochemistry of Marion and Prince Edward Islands, Southern Ocean: Magma chamber processes and source region characteristics. *J. Volcanol. Geotherm. Res.* **2012**, *223-224*, 11-28.

12. Weis, D.; Frey, F.A.; Giret, A.; Cantagrel, J.M. Geochemical characteristics of the youngest volcano (Mount Ross) in the Kerguelen Archipelago: inferences for magma flux, lithosphere assimilation and composition of the Kerguelen plume. *J. Petrol.* **1998**, *39*, 973-994.

13. Barling, J.; Goldstein, S.L.; Nicholls, I.A. Geochemistry of Heard Island (Southern Indian Ocean): Characterization of an enriched mantle component and implications for enrichment of the sub-Indian ocean mantle. *J. Petrol.* **1994**, *35*, 1017-1053.

14. Doucet, S.; Weis, D.; Scoates, J.S.; Debaille, V.; Giret, A. Geochemical and Hf-Pb-Sr-Nd isotopic constraints on the origin of the Amsterdam-St. Paul (Indian Ocean) hotspot basalts. *Earth Planet. Sci. Lett.* **2004**, *218*, 179-195.

15. Harmon, R.S.; Barreiro, B.A.; Moorbath, S.; Hoefs, J.; Francis, P.W.; Thorpe, R.S.; Deruelle, B.; McHugh, J.; Viglino, J.A. Regional O-, Sr-, and Pb-isotope relationships in late Cenozoic calc-alkaline lavas of the Andean Cordillera. *J. Geol. Soc. London* **1984**, *141*, 803-822.

16. Stern, C.R.; Kilian, R. Role of the subducted slab, mantle wedge and continental crust in the generation of adakites from the Andean Austral Volcanic Zone. *Contrib. Mineral. Petrol.* **1996**, *123*, 263-281.
